# Leveraging technology to improve care: a real-time electronic dashboard within an anticoagulation stewardship service to optimise anticoagulant safety

**DOI:** 10.1007/s11239-026-03277-5

**Published:** 2026-04-17

**Authors:** Emma Jane Leitinger, Nadishani Ratnayake, Simone Taylor, Hiu Lam Agnes Yuen, Jasmine Singh, Charithani Keragala, Kay Htun, Nicholas Ling, Katherine Wilson, Tania Duncan, Zane Kaplan, Mark Sheppard, Susan Brown, Sanjeev Chunilal, Anastasios Nalpantidis

**Affiliations:** 1 Department of Haematology, Level 3, Monash Health, Monash Medical Centre, 246 Clayton Road, Melbourne, VIC 3168 Australia; 2 School of Clinical Sciences at Monash Health, Monash University, Clayton Campus, Melbourne, VIC Australia; 3 Department of Pharmacy, Monash Health, Melbourne, VIC Australia; 4 School of Public Health and Preventive Medicine, Monash University, Melbourne, VIC Australia; 5 Australian Centre for Blood Diseases, Melbourne, VIC Australia; 6 Business Intelligence, Monash Health, Melbourne, VIC Australia; 7 Department of Haematology, Alfred Health, Melbourne, VIC Australia

**Keywords:** Medication errors, Anticoagulation Stewardship, Anticoagulants, Patient safety, Electronic dashboards, Clinical decisionsupport systems

## Abstract

Anticoagulants are high-risk medications associated with significant morbidity and mortality when used inappropriately. Electronic medical records, laboratory results, and medication data can be combined into electronic “dashboards”, providing a novel way to screen patients prescribed anticoagulants for potential errors. To describe and evaluate the impact of an efficient electronic dashboard within an Anticoagulation Stewardship service able to identify and facilitate proactive intervention for at-risk patients prescribed anticoagulants. Retrospective audit of adult inpatients receiving anticoagulants between 8th November 2022 and 31st January 2024 in a large multicentre tertiary hospital network. The dashboard was used daily to screen all hospital inpatients prescribed anticoagulants. Prescribing errors and bleeding risk factors were identified, and recommendations were made to treating teams. Cases were subclassified as “high-risk” or “standard-risk” based on the prescribing error and likelihood of harm. Combined clinically relevant non major bleeding (CRNMB) and major bleeding (MB) at 30 days post-intervention. Approximately 10,500 prescribing events were screened, 718 (6.9%) requiring a change to therapy in 611 patients. Of these patients, 292 (48%) were sub-classified as “high-risk”, and the remainder were “standard-risk” (*n* = 319, 52%). “High-risk” patients demonstrated higher rates of MB/CRNMB compared to those deemed “standard-risk” (7.2% vs. 3.1%, *p* = 0.022). This real-time dashboard enables rapid screening and identification of inpatients at risk of anticoagulant-related bleeding, allowing for timely intervention to reduce harm.

**Graphical abstract:**

**Figure:**
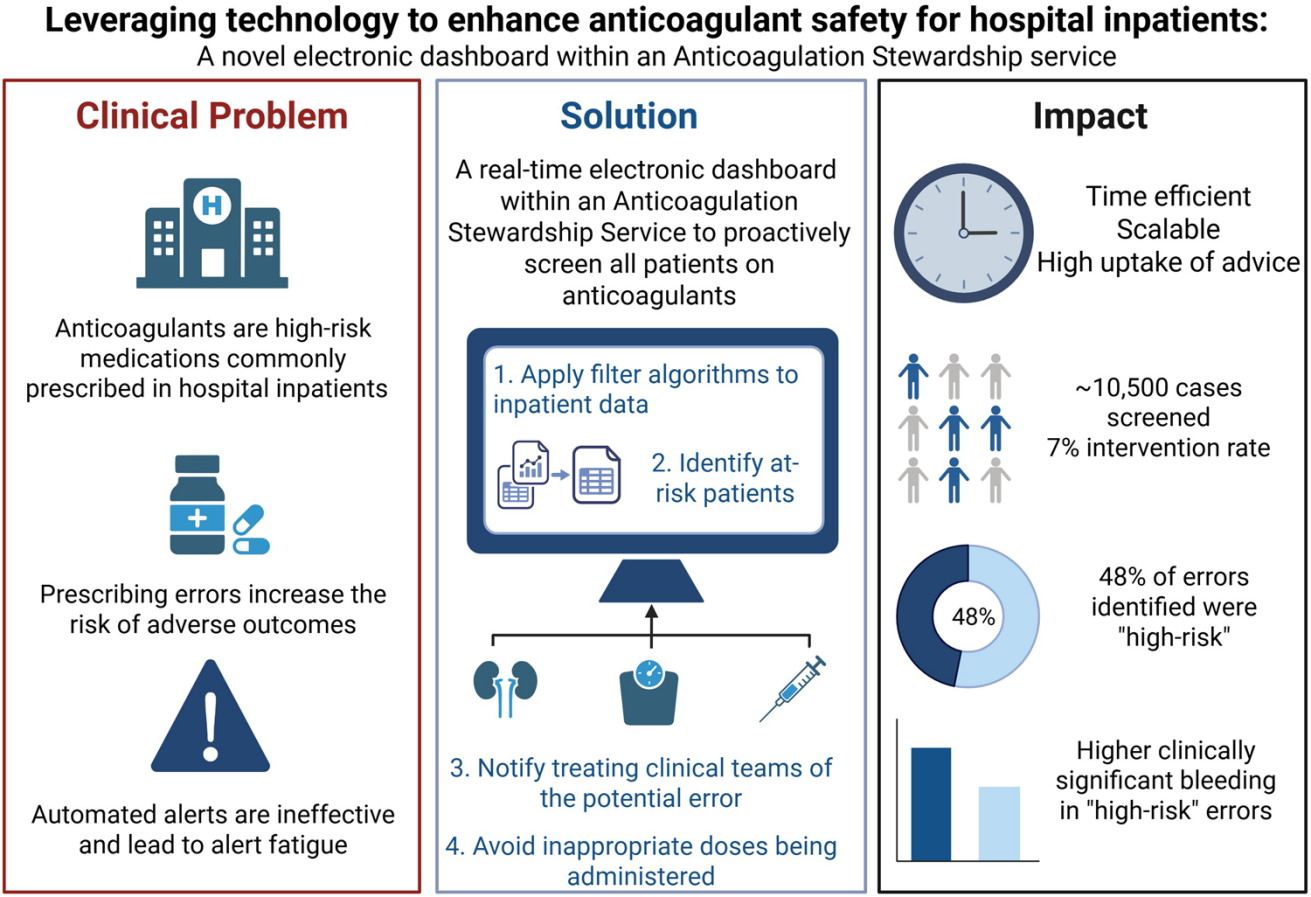

**Supplementary Information:**

The online version contains supplementary material available at 10.1007/s11239-026-03277-5.

## Key Points

A real time, electronic anticoagulation dashboard embedded within an Anticoagulation Stewardship (ACS) service enabled rapid screening of high patient numbers to identify and intervene on potential prescribing errors.

Nearly half of cases requiring intervention had high-risk prescribing errors, and demonstrated significantly higher 30 day clinically relevant bleeding rates (7.2% vs 3.1%, p = 0.022).

Timely ACS interventions prevented administration of inappropriate anticoagulant doses in ~30% of cases, with high clinician acceptance of advice.

Future directions: Expansion of the dashboard to incorporate medication interaction checks and dynamic tracking of changes in key laboratory results could further enhance predictive accuracy and allow development of targeted alerting systems.

**Supplementary Information:**

The online version contains supplementary material available at 10.1007/s11239-026-03277-5.

## Background

 Anticoagulants are widely used, high-risk medications, with the major side effect of bleeding. Since the introduction of the direct oral anticoagulants (DOACs), prescribing of these therapies has continued to increase [1]. Anticoagulants are implicated in over 20% of Emergency Department presentations related to medication adverse events, and account for 8.3% of inpatient prescribing errors, up to one third of which are due to duplicate prescribing [2–6]. Bleeding rates in inpatients prescribed anticoagulants are poorly defined, ranging from 2.2% to 15.8% [7–9]. Bleeding risk factors include advanced age, female sex, hypertension, recent procedures, renal impairment, and concurrent antiplatelet therapies [10–12]. These need to be balanced with thrombotic risk to provide individualised patient care [13–16].

The widespread use of electronic prescribing, medical records, and laboratory results, allows for safety checks, such as automated alerts for duplicate prescribing of medications or high risk interactions. However, “alert fatigue”; where clinical staff dismiss the automated alert message, limits their efficacy [17–19]. Therefore, there remains an unmet need to better oversee prescribing of these high-risk therapies in the inpatient setting. Electronic decision support tools, or “dashboards”, are a strategy to easily screen patients prescribed anticoagulants, facilitating review for therapy appropriateness [20–22]. Embedding these tools within an Anticoagulation Stewardship (ACS) service has yielded promising results. This has been described in the outpatient setting, allowing for prioritisation of patients for review, improved prescribing practices, and higher clinic attendance and medication adherence [23–26]. A key limitation is the use of retrospective data rather than updating in real time, which is unable to address the needs of hospital inpatients. Daniel et al. describe a “near real-time” system for pharmacists which was able to screen inpatients prescribed anticoagulants across 31 hospitals, resulting in a 40.6% reduction in medication adverse events. While encouraging, the absolute difference in adverse events was only 0.28% (0.69% to 0.41%), therefore, systems better able to identify at-risk patients to target interventions to are needed [27].

We report here the design, implementation and initial review of a novel, real-time, electronic dashboard embedded within an ACS service to monitor prescribing safety. This tool allows rapid screening and identification of hospital inpatients prescribed anticoagulant therapies deemed to be at increased risk of anticoagulation associated bleeding.

## Methods

### Objectives and outcome measures

To describe the design and implementation of a novel electronic dashboard within an ACS service, including patient characeristics of those identified as being at-risk of anticoagulant-related harm, types of interventions undertaken by the ACS team, 30-day outcomes for patients following ACS intervention, and rates of hospital-coded bleeding events secondary to anticoagulants pre- and post-implementation. The primary outcome at 30 days post-ACS intervention was clinically significant bleeding, defined as clinically relevant non major bleeding (CRNMB) and major bleeding (MB) as per International Society of Thrombosis and Haemostasis (ISTH) defitions [28]. Secondary outcomes were all bleeding events; a composite of bleeding, thrombosis, death and unplanned readmission; length of stay; and number of inappropriate anticoagulant doses received based on current prescribing guidelines. Bleeding events were independently adjudicated by a panel of haematologists and pharmacists.

### Anticoagulation stewardship service and electronic dashboard design

Monash Health is the largest tertiary healthcare network in Melbourne, Australia, with a catchment of 1.8 million residents. It comprises approximately 2000 inpatient beds across 7 campuses, with 4 emergency departments and 4 intensive care units. An ACS program was implemented in November 2022 following several sentinel events related to anticoagulant prescribing errors and associated bleeding. The service operates Monday to Friday 8am-4:30pm, staffed by 2.0 full-time equivalent (FTE) pharmacists (1 lead pharmacist and 1 rotating pharmacist), 0.8 haematologist FTE and 0.8 thrombosis nursing FTE.

**Fig. 1:**
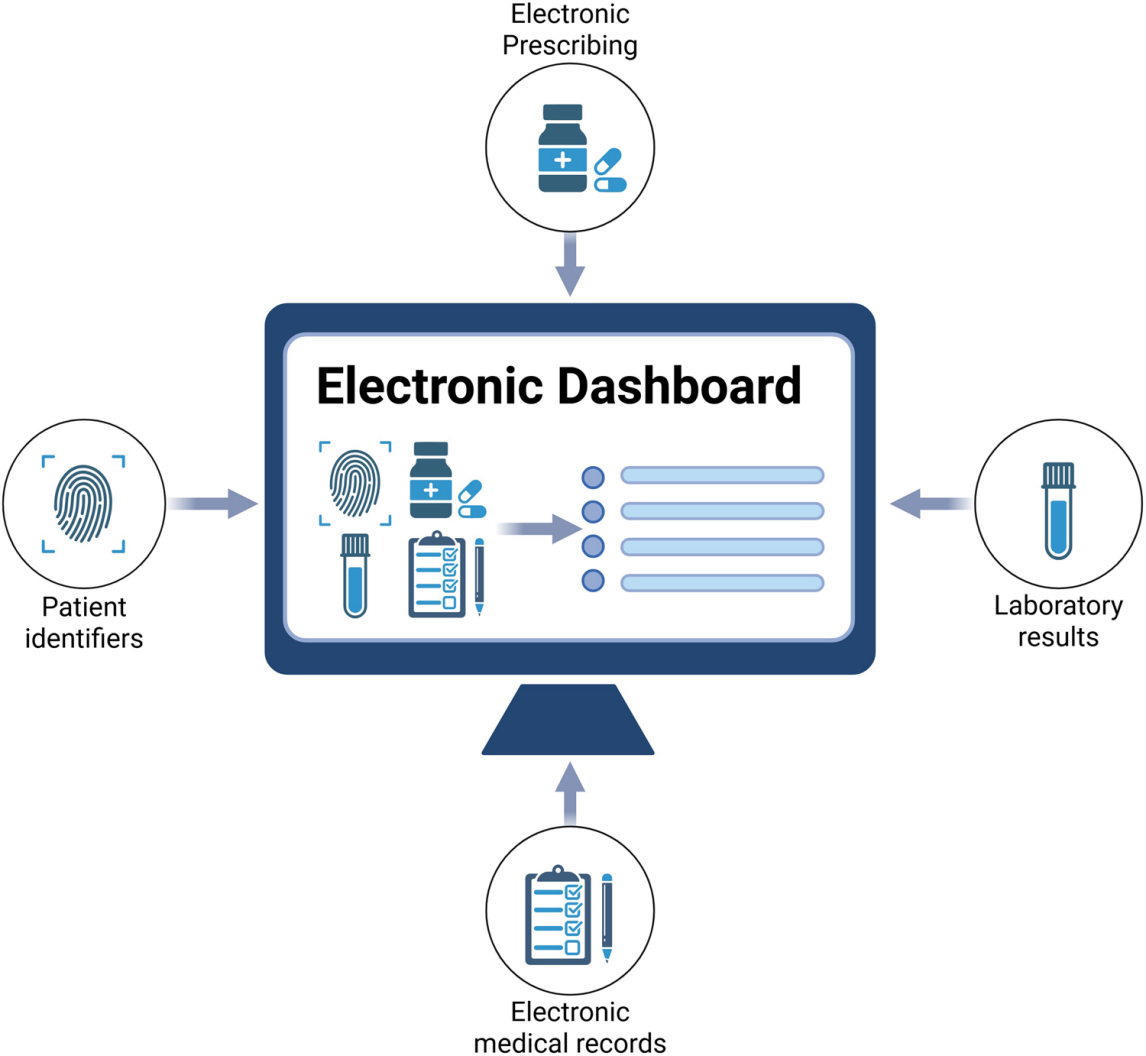
Dashboard design schematic

To facilitate implementing this program across all inpatient sites, an electronic dashboard was designed in conjunction with the hospital business intelligence team (BI). This tool integrates multiple hospital electronic systems, collating and tabulating patient information including characteristics (e.g. age, weight), prescribed anticoagulants / antiplatelet agents, and relevant laboratory results (platelet count, renal function) (Fig. 1). The ACS pharmacists then apply the filter algorithms to interrogate the data, and identify adult inpatients with potential prescribing errors, e.g. inappropriate dosing for renal function, or duplicate anticoagulant therapy. Filter variables were predefined using available literature, sentinel event data, and expert consensus (Table 1).

Dashboard-identified patients were then screened by the ACS team to determine whether intervention was required, providing recommendations and advice to the treating clinical teams to improve prescribing safety and appropriateness of anticoagulant use. Cases requiring intervention were subclassified as “high-risk” if they met the criteria outlined in Table 1, or “standard-risk” if they did not meet these criteria. High-risk cases were followed up the same day to ensure the identified issue was corrected, minimising the chance of any inappropriate anticoagulant doses being administered. This dashboard updates hourly and used by the ACS team daily on weekdays to review all hospital inpatients prescribed anticoagulants. Any prescribing events occurring outside of these hours were reviewed the following business day.

Figure 1 Schematic outlining the dashboard design incorporating relevant patient information. Data are collated and displayed in a table which updates depending on the filter algorithms applied. This allows rapid screening of all patients prescribed anticoagulants to assess for appropriateness.

**Table 1:** Dashboard filter criteria and risk stratification criteria

*Filters to identify “at-risk” patients*
1. Medication errors a. ≥ 2 anticoagulants prescribed concurrently 2. Patient characteristics a. Extremes of weight (< 40 kg, > 130 kg) b. Advanced age (≥ 80 years) with renal impairment (eGFR < 50mL/min) 3. Abnormal laboratory results a. eGFR < 40 mL/min b. Platelets < 50 × 10^9^/L c. INR > 4.0 d. aPTT > 100 e. All anti-Xa levels 4. High-risk medications a. Heparin infusions b. Non-standard anticoagulants (e.g. bivalirudin, fondaparinux, danaparoid, argobatran, dalteparin)
*High risk criteria*
1. ≥ 2 anticoagulants charted concurrently^a^ 2. Platelets < 50 × 10^9^/L 3. eGFR < 30 mL/min on inappropriate dose therapeutic anticoagulation 4. Supratherapeutic anti-Xa^b^

mL/min = millilitres per minute; eGFR = estimated glomerular filtration rate; INR = international normalised ratio; APTT = activated partial thromboplastin time; anti-Xa = anti-factor-Xa

^a^Excluding appropriate warfarin bridging with enoxaparin

^b^Anti-Xa levels above “on-therapy” range defined by local guidelines for dosing regimens at peak timing, or within “on-therapy” range at trough timing

### Study design

Retrospective audit of all adult inpatients identified by the dashboard who required intervention, from date of implementation (8th November 2022) to 31st January 2024. Patient characteristics were collected prospectively, including demographics, medications, laboratory results, dashboard algorithms, interventions, and time per case reviewed. If a patient was identified on more than one occasion, only initial review was included for analysis. If patients were identified by multiple dashboard filter criteria, the most clinically significant criterion was assigned for analysis. Data were not collected for patients not requiring intervention. ACS recommendations were classified as dose adjustment (alterations to dosing or withholding a dose), therapy change / cessation (change of anticoagulant or ceasing an anticoagulant e.g. if 2 concurrent anticoagulants were prescribed), drug monitoring (e.g. recommending medication levels), and “other” if they did not fall into these categories. Intervention cases were followed up via chart review to assess whether ACS recommendations were implemented, 30-day outcomes (clinically significant bleeding; composite of all bleeding, thrombosis, unplanned readmission and death), number of inappropriate doses of anticoagulant received (or days of inappropriate therapy for infusions), and length of stay. Finally, medical record data for rates of haemorrhage secondary to circulating anticoagulants (hospital acquired complication (HAC) rates as per ICD-10-AM coding) were assessed over a 14 month period before and after implementation [29].

### Statistical analysis and sample size calculations

Patient characteristics were reported as frequency and percentage for categorical variables, and median and interquartile range (IQR) were used for continuous variables. Bleeding outcomes were compared using Chi Square or Fishers exact test for categorical variables, and Mann-Whitney-U test for continuous variables. Planned subgroup analysis was performed comparing bleeding between high-risk patients and standard-risk patients. Exploratory post-hoc analysis was performed to identify associations between the number of inappropriate medication doses received and rates of bleeding. Statistical significance was determined with a two tailed p value less than 0.05. Analysis was performed using IBM SPSS 29.0 and Microsoft Excel.

No sample size calculation was undertaken as this was an exploratory review of the first 14 months of implementation. Therefore, all cases who required intervention within this time were included. The study was approved by the local ethics review board (QA/104693/MonH-2023-405505).

## Results

### Case identification and interventions

**Fig. 2:**
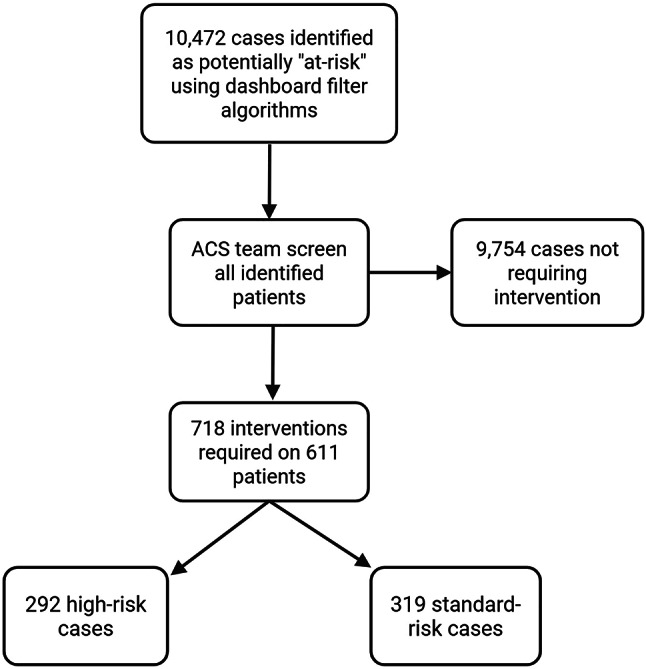
Case identification breakdown

A total of 10,472 prescribing events were identified over 14 months using the prespecified filter algorithms, 718 of which required intervention. This equates to 34 prescribing events screened daily with a 6.9% intervention rate. These 718 events occurred in 611 individual patients, 292 (47.8%) of which were classified as “high-risk” (Fig. 2). Prescribing errors accounted for the majority (413/611, 67.6%) of patients requiring intervention, one-third of which were inappropriate duplicate prescribing of anticoagulants.

### Patient characteristics

Patient characteristics are outlined in Table 2. There was equal gender distribution, and a median age of 75 years (IQR 60–85 years), with moderate renal impairment (eGFR 44mL/min and 47mL/min in high-risk vs. standard risk cases). Concomitant antiplatelet use was 13.4%. Enoxaparin was the most prescribed anticoagulant (145, 64.3%), followed by apixaban (103, 17.3%), then warfarin (57, 9.3%), and under 5% for other agents. Indications for anticoagulation were atrial fibrillation (AF, 28.5%), venous thromboembolism treatment (VTE, 23.7%), and VTE prophylaxis (VTEP, 35.2%). Within the high-risk group, almost half (124 / 292, 48.6%) were anticoagulated for AF, whereas in the standard risk group the most common indication was VTE prophylaxis (135 / 319, 42.3%).

**Table 2:** Patient characteristics

Parameter	High-risk cases (*n* = 292)	Standard risk (*n* = 319)
*Sex (%)*		
Male Female	153 (52.4) 139 (47.6)	184 (57.7) 135 (42.3)
Median age (years) (IQR)	77 (65.3, 85.8)	72 (55, 84)
Median weight (kg) (IQR)^a^	77.3 (63.6, 93)	85.6 (68.7, 150.5)
Median eGFR (mL/min) (IQR)^b^	44 (23, 80)	47 (30, 90)
Median platelet count (x10^9^/L ) (IQR)^c^	204 (120.5, 290)	252 (182.5, 323)
Antiplatelet agents (%)	34 (11.6)	48 (15.0)
*Primary medication (%)*		
Enoxaparin Apixaban Rivaroxaban Dabigatran Warfarin Heparin Other	171 (58.6) 92 (31.5) 15 (5.1) 4 (1.4) 5 (1.7) 5 (1.7) 0 (0)	222 (69.6) 14 (4.4) 7 (2.2) 1 (0.3) 52 (16.3) 21 (6.6) 2 (0.6)
*Anticoagulation indication (%)*		
VTE treatment AF VTE prophylaxis ATE Mechanical valve Other	70 (24.0) 124 (48.6) 80 (27.4) 12 (4.1) 2 (0.7) 4 (1.4)	75 (23.5) 50 (15.7) 135 (42.3) 34 (10.7) 20 (6.3) 5 (1.6)
Procedure within 30 days of ACS intervention	73 (25.0)	94 (29.5)
Bleeding prior to ACS review^d^ (%)	42 (14.4)	56 (17.6)
*Site of bleeding*		
ICH GIT GUT Mucocutaneous Surgical site Retroperitoneal Other	2 (0.7) 14 (4.8) 4 (1.4) 4 (1.4) 5 (1.7) 10 (3.4) 3 (1.0)	2 (0.6) 12 (3.8) 12 (3.8) 8 (2.5) 4 (1.4) 8 (2.5) 10 (3.1)

IQR = interquartile range; kg = kilograms; eGFR = estimated glomerular filtration rate; mL/min = millilitres per minute; ICH = intracranial haemorrhage; GIT = gastrointestinal tract; GUT = genitourinary tract; VTE = venous thromboembolism; AF = atrial fibrillation; ATE = arterial thromboembolism

^a^19 missing weight

^b^11 missing eGFR

^c^5 missing platelet count

^d^Documented bleeding during the same inpatient admission the patient was identified by the dashboard and required intervention from the ACS team, but occurring prior to ACS intervention

**Fig. 3:**
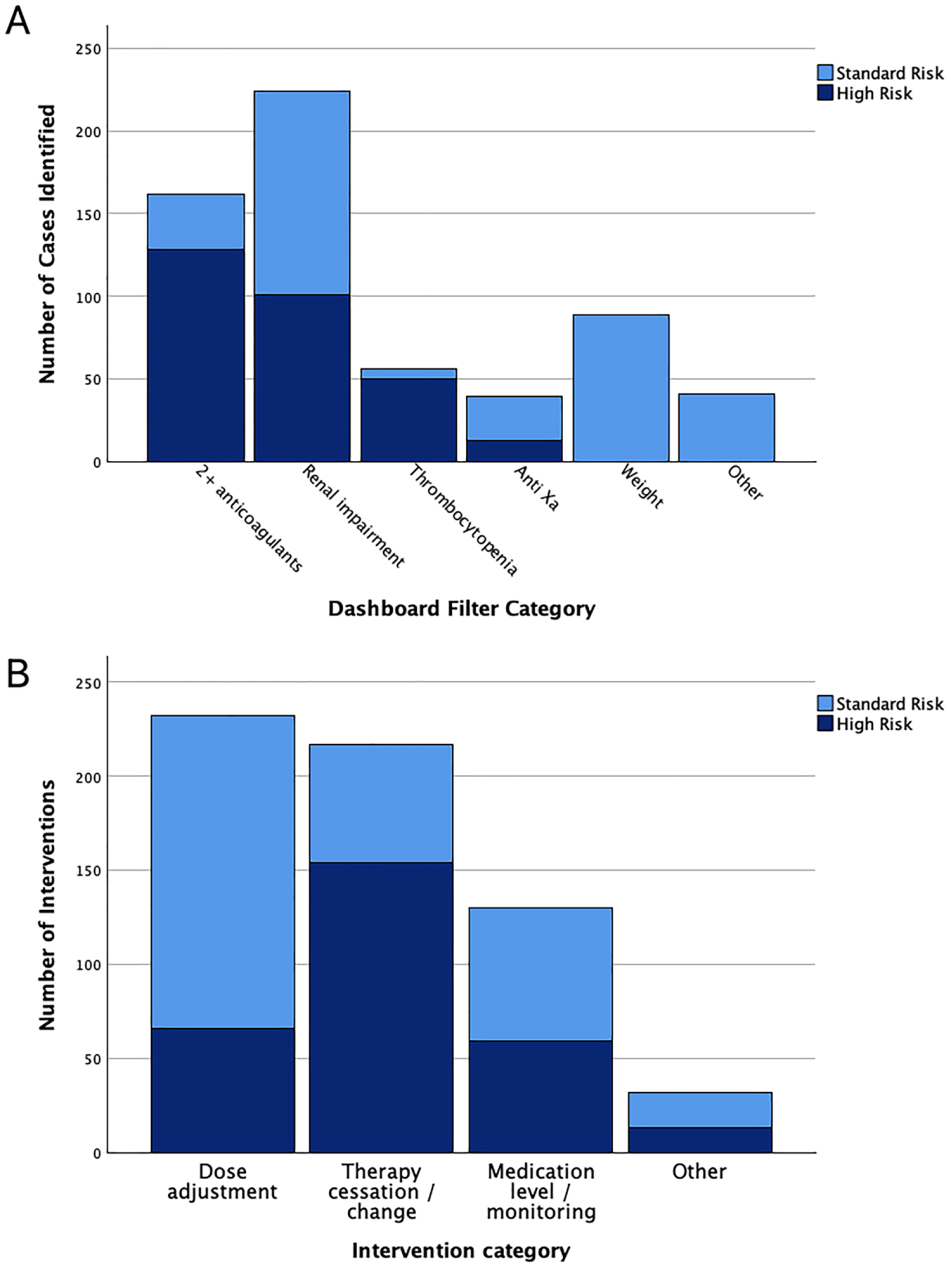
Case identification and interventions by dashboard filter criteria and risk category. **A**. Number of patients identified by the dashboard algorithms with high-risk cases denoted in dark blue, and standard-risk in light blue. **B**. Interventions carried out by the ACS team on patients identified in Fig. 3A divided into high-risk (dark blue) and standard-risk (light blue)

Renal impairment was the most common filter algorithm which identified at-risk cases (224 / 611, 36.7%), followed by duplicate prescribing (162 / 611, 26.5%), and extremes of bodyweight (89 / 611, 14.6%) (Fig. 3A). Most high-risk cases were inappropriately charted for 2 concurrent anticoagulants (128 / 292, 43.8%), of which 51.6% (66 / 128) were prescribed a therapeutic and prophylactic anticoagulant (most commonly therapeutic DOAC and prophylactic enoxaparin), 49 (38.3%) prescribed low dose DOAC and prophylactic enoxaparin or duplicate enoxaparin prophylaxis, and 13 (10.2%) were prescribed full therapeutic doses of both anticoagulants. Remaining high-risk cases comprised renal impairment with inappropriate anticoagulant doses (101 / 292, 34.6%), thrombocytopenia < 50 × 10^9^/L (50 / 292, 17.1%), and supra-therapeutic anti-Xa levels (13 / 292, 4.5%). Moderate renal impairment with concurrent anticoagulant therapy was the most common category within the standard risk group (161 / 319, 50.5%), followed by those at extremes of bodyweight (106 / 319, 33.2%). Those in the “other” category included small numbers of patients prescribed intravenous anticoagulants (heparin, bivalirudin), and those with prolonged APTT or INR.

The most common intervention was dosage adjustment (232 / 611, 37.9%), 131 (56.5%) of which were dose reductions, primarily due to renal impairment (Fig. 3B). Changing or ceasing anticoagulant therapy was recommended in 217 cases (35.5%) overall, 71.0% (154 / 217) of which were in the high-risk group, reflecting dual anticoagulant prescribing. 21.2% of cases were recommended to assess medication levels or other monitoring parameters, and 5.2% of recommendations were classed as “other”.

Over 85% of interventions were completed within 15 min from the time of case identification to informing the treating unit of the recommendation. Uptake of recommendations was high at 82%, 96.8% of which were implemented within 24 h. This avoided administration of any inappropriate anticoagulant doses in 29.8% of total cases (182 / 611), and 42.5% of high-risk cases (124 / 292). Recommendations not being implemented was most frequently due to resolution of the issue identified by the dashboard, discharge or interhospital transfer, and change in direction of care (palliation).

### 30-day outcomes

There were 31 clinically relevant bleeding events following ACS intervention, 21 occurring in the high-risk group and 10 in the standard-risk group (7.2% vs. 3.1%, *p* = 0.022). This included 10 major bleeds, 4 of which were fatal, and 21 clinically relevant non-major bleeds. The majority (8/10) of major bleeds, and 3 of the 4 fatal bleeds, occurred in the high-risk group. Additional information in appendix Table 1.

The composite endpoint of bleeding, thrombosis, unplanned readmission, or death occurred in 190 (31.1%) of the 611 patients, 75 (39.3%) of which were attributable to bleeding and/or thrombosis. This included a total of 53 bleeding events (including minor bleeding), and 22 thrombotic events. Of the 53 bleeding events following ACS intervention, 29 had documented bleeding during the same inpatient admission preceding ACS review, 16 of which reccurred at the same site following ACS intervention (predominantly gastrointestinal and genitourinary). The majority of thrombotic events were arterial, 3 of which may have occurred prior to ACS intervention. Only 7 readmissions and 8 deaths were related to bleeding or thrombosis. All readmissions due to bleeding occurred in the high-risk group, 2 of which were fatal (1 intracranial and 1 gatrointestinal bleed). There were 4 deaths attributable to thrombosis, all of which had occurred prior to ACS intervention. There was no significant difference in length of stay between patient groups, nor those with bleeding events. Median follow-up was 15 days (IQR 5, 35) following ACS intervention. See Table 3 for further detail.

**Table 3:** 30-day patient outcomes post ACS intervention

Outcomes	High risk (%) *n* = 292	Standard risk (%) *n* = 319	*p* value
*Primary outcome*			
Clinically significant bleeding (CRNMB + MB) MB CRNMB	21 (7.2) 8 (2.7) 13 (4.5)	10 (3.1) 2 (0.6) 8 (2.5)	0.022 0.054 0.63
*Site of bleeding*			
ICH GIT GUT Mucocutaneous Surgical site Retroperitoneal Other	1 (0.3) 8 (1.7) 3 (1.0) 1 (0.3) 2 (0.7) 1 (0.3) 5 (1.7)	0 (0) 2 (0.6) 3 (0.9) 2 (0.6) 2 (0.6) 0 (0) 1 (0.3)	0.48 0.054 1.0 1.0 1.0 0.48 0.198
*Secondary outcomes*			
Total bleeding events	31 (10.6)	22 (6.9)	0.114
Composite endpoint			
*(Bleeding*,* thrombosis*,* death and unplanned readmission)* Bleeding / Thrombosis related	99 (33.9) 44 (15.1)	90 (28.2) 32 (10.0)	0.108 0.010
New or recurrent thrombosis (total) ATE^a^ VTE	12 (4.1) 9 (3.1) 3 (1.0)	10 (3.1) 6 (1.9) 4 (1.3)	0.53 0.34 1.0
Readmissions (total) Related to bleeding / thrombosis Unrelated to bleeding / thrombosis	43 (14.7) 6 (2.1) 37 (12.7)	36 (11.3) 1 (0.3) 35 (11.0)	0.23 0.059 0.52
Deaths (total) Related to bleeding / thrombosis Unrelated to bleeding / thrombosis	34 (11.6) 3 (1.0) 31 (10.6)	41 (12.9) 5 (1.6) 36 (11.2)	0.71 1.0 0.79
Length of stay (days) (IQR)	16 (7, 37)	19 (8, 46)	0.66
Total number of inappropriate anticoagulant doses received (median, IQR)	1 (0, 3)	2 (1, 3)	0.61
0–1 inappropriate doses (%) 2 or more inappropriate doses (%) Unable to determine (%)	162 (55.4) 97 (33.2) 33 (11.3)	119 (37.3) 135 (42.3) 65 (20.4)	< 0.001 0.021 NA

MB = major bleeding; CRNMB = clinically relevant non-major bleeding; ICH = intracranial haemorrhage; GIT = gastrointestinal tract; GUT = genitourinary tract; VTE = venous thromboembolism; ATE = arterial thromboembolism; NA = not assessed

Readmission = unplanned (excluding dialysis patients and other planned readmissions)

^a^5 intracardiac thromboses, 8 strokes, of which 3 had possibly occurred prior to ACS intervention and 2 were post-procedures, 1 ischaemic limb

Review of hospital coding data for bleeding secondary to anticoagulants confirmed that all events related to prescribing errors would have been identified by the dashboard filter algorithms. There was a 31% reduction in total events following ACS service implementation (39 at baseline, 27 post-ACS), and approximately a 50% reduction in potentially preventable events (15 pre-ACS, 8 post-ACS).

Post-hoc analysis demonstrated a lower rate of bleeding in patients who received fewer inappropriate anticoagulant doses (0 to 1 doses administered) compared to those receiving 2 or more inappropriate doses (8.5% vs. 15.4%, *p* = 0.021) (see appendix Fig. 1 for further detail). A similar trend was seen for those experiencing clinically significant bleeding (CRNMB/MB), and the high-risk patients, however this did not reach statistical significance (appendix Table 2).

## Discussion

An anticoagulation stewardship service was successfully implemented in a large, tertiary, multisite hospital network incorporating a novel electronic dashboard to screen high patient numbers in real-time. This allowed for proactive intervention to correct potential prescribing errors, avoiding inappropriate medication administration in 30% of identified cases. Clinically significant bleeding rates were greater in high-risk patients (7.2% vs. 3.1%), and exploratory analysis suggests that early intervention to reduce administration of inappropriate anticoagulant doses was associated with a lower risk of bleeding.

Despite existing inbuilt electronic prescribing alerts and regular education, serious prescribing errors were still seen in ~ 4% of hospital inpatients, one third of which were due to inappropriate duplicate prescribing. This supports previously reported rates in the literature and highlights the need for systems able to identify and correct these errors such as electronic dashboards to combat “alert fatigue” [18, 19, 30]. The majority of published dashboards are tailored to the outpatient setting, and are not updated frequently enough to reflect changes in acute hospital inpatients [22]. The near real-time model described by Daniel et al. tried to address this, and successfully reduced medication-related adverse events by 40% [27]. However, the low event rates resulted in a number needed to treat (NNT) of 357. While these results were statistically significant, they highlight the need to better target at-risk patients to optimise impact and resource allocation. By comparison, our model had a higher intervention rate per number of cases screened, and higher rates of bleeding which may suggest we were able to identify patients at higher risk of harm.

Baseline bleeding rates in the at-risk inpatient population identified by our dashboard are unknown as targeted screening and identification has not previously been performed. Reported inpatient bleeding rates are highly variable, although our rate appears comparable with Dreijer et al. who described a demographically similar patient population to our study [11]. Interestingly, most bleeding events identified were procedure-related, with a rate of only 2.2% in non-surgical patients. Conversely, the majority of our bleeding events were not related to procedures, which may suggest we are able to identify an at-risk, non-surgical patient population. The difference in clinically significant bleeding rates supports the stratification of patients into high-risk and standard-risk groups, which assists in prioritising patients who may require closer monitoring and follow-up. Current filter algorithms would have identified all prescribing errors from historic HAC events, thus would have allowed for proactive intervention which may have prevented some events. This is supported by the reduction in HAC events and trend towards less bleeding in patients administered fewer inappropriate anticoagulant doses, though further evaluation is required. High uptake of advice in a timely manner further supports the acceptability and utility of the service.

The high rates of readmission and death due to other causes in our study may reflect an older, comorbid patient population, who are more likely to have risk factors for both bleeding and thrombosis. Importantly, thrombotic events were low, and the majority were related to atrial fibrillation or underlying cardiac disease.

While this dashboard appears to facilitate identification and intervention for patients at risk of anticoagulant-related harm, there are important limitations. Filter algorithms are reliant on electronic records updating regularly, and treating teams accurately recording parameters such as weight. Data on medication interactions or a significant change in key variables (e.g. renal function, platelet count) are not available in the described dashboard, though are being developed. Other bleeding risk factors such as comorbidities cannot easily be incorporated into an automated screening system, thus manual record review is still required for some cases. Service model limitations include the inability to screen for potential prescribing errors outside of service hours, therefore inappropriate doses may still be administered if the treating teams do not identify the error. The retrospective assessment of outcome data relies on availability and accuracy of information within patient records. Finally, the lack of baseline bleeding and thrombotic outcomes in an equivalent patient cohort hinders direct assessment of service efficacy and cost benefit.

## Conclusions

Anticoagulant prescribing errors remain common despite in-built alerts in electronic medical records, thus additional safeguards are required. This novel electronic dashboard allows for rapid screening of high patient numbers by a small ACS team, identifying and intervening on cases at risk of anticoagulant-related harm to minimise bleeding risk. Our real-time model appears well-suited to hospital inpatients, and can help address distance and specialist access barriers, promoting effective resource allocation.

## Supplementary Information

Below is the link to the electronic supplementary material.

Supplementary Material 1

## Data Availability

All data supporting the findings of this study are available within the paper and its Supplementary Information. Further data can be provided on request.
