# Supplementary material for: Leveraging technology to improve care: a real-time electronic dashboard within an anticoagulation stewardship service to optimise anticoagulant safety
Source: J Thromb Thrombolysis. 2026 Apr 17;59(6):1618–27. doi: 10.1007/s11239-026-03277-5 (PMC13447389; doi:10.1007/s11239-026-03277-5)
Supplement: Supplementary file 1 [file 11239_2026_3277_MOESM1_ESM.docx]

**Appendix**

**Table 1. Patient characteristics for clinically relevant non-major bleeding and major bleeding cases**

| **Case characteristics** | **MB**  **n = 10** | **CRNMB**  **n = 21** | **Total cases (n=611)** |
| --- | --- | --- | --- |
| **Sex (%)**  Male  Female | 6 (60)  4 (40) | 11 (52)  10 (48) | 337 (55.1)  274 (44.9) |
| **Median age (years)** | 78 | 75 | 75 |
| **Median weight (kg)** | 75 | 80 | 80 |
| **Median eGFR (mL/min)** | 36 | 37 | 39 |
| **Median platelet count (**x 10^9^/L) | 205 | 228 | 229 |
| **Antiplatelet therapy (%)** | 1 (10) | 2 (10) | 82 (13.4) |
| **Anticoagulation indication (%)**  VTE  AF  VTE prophylaxis  ATE  Mechanical valve  Other | 0 (0)  6 (60)  2 (20)  1 (10)  0 (0)  1 (10) | 7 (33)  7 (33)  5 (24)  2 (10)  0 (0)  0 (0) | 145 (23.7)  174 (28.5)  215 (35.2)  46 (7.5)  22 (3.6)  9 (1.5) |
| **Site of bleed (%)**  ICH  GIT  GUT  Mucocutaneous  Surgical/procedural  Retroperitoneal  Other | 1 (10)  5 (50)  0 (0)  0 (0)  1 (10)  1 (10)  2 (20) | 0 (0)  5 (24)  6 (29)  3 (14)  3 (14)  0 (0)  3 (14) | - |
| **Death due to bleeding** | 4 | 0 | - |
| **Bleeding prior to ACS review**^a^ **(%)** | 2 (20%) | 11 (53%) | 84 (14%) |
| **High risk**  ≥2 anticoagulants  eGFR<30  Thrombocytopenia  Supratherapeutic anti-Xa | 8  3  3  1  1 | 13  7  5  1  0 | 292 (48%)  128 (46%)  101 (21%)  50 (9%)  13 (2%) |

kg = kilograms; eGFR = estimated glomerular filtration rate; mL/min = millilitres per minute; MB = major bleeding; CRNMB = clinically relevant non-major bleeding; eGFR = estimated glomerular filtration rate; ICH = intracranial haemorrhage; GIT = gastrointestinal tract; GUT = genitourinary tract; VTE = venous thromboembolism; AF = atrial fibrillation; ATE = arterial thromboembolism.

^a^Documented bleeding during the same inpatient admission the patient was identified by the dashboard and required intervention from the ACS team, but occurring prior to ACS intervention.

**Figure 1. Number of inappropriate doses received (or days at inappropriate dosing for infusions)**

Number of doses of anticoagulant therapy administered to patients requiring ACS intervention deemed inappropriate based on local and best-practice guidelines.

**Table 2. Bleeding risk by number of inappropriate doses received**

|  | **Number of doses received** | | **p value** |
| --- | --- | --- | --- |
|  | **0 to 1 doses** | **2 or more doses** |  |
| **All cases (n=718)** | | | |
| Any bleeding (%) | 7.8 | 13.4 | **0.021** |
| No bleeding (%) | 92.2 | 86.6 |  |
| CRNMB+MB (%) | 4.3 | 7.3 | 0.096 |
| No CRNMB+MB (%) | 5.7 | 92.7 |  |
| **High risk (n=292)** | | | |
| Any bleeding (%) | 9.3 | 17.5 | 0.092 |
| No bleeding (%) | 90.7 | 82.5 |  |
| CRNMB+MB (%) | 6.2 | 11.3 | 0.069 |
| No CRNMB+MB (%) | 93.8 | 88.7 |  |
| **Standard risk (n=319)** | | | |
| Any bleeding (%) | 5.9 | 10.4 | 0.106 |
| No bleeding (%) | 94.1 | 89.6 |  |
| CRNMB+MB (%) | 1.7 | 4.4 | 0.45 |
| No CRNMB+MB (%) | 98.3 | 95.6 |  |
